# The Aromatase Gene *(CYP19A1)* Variants and Circulating Hepatocyte Growth Factor in Postmenopausal Women

**DOI:** 10.1371/journal.pone.0042079

**Published:** 2012-07-25

**Authors:** Jennifer H. Lin, Marc J. Gunter, JoAnn E. Manson, Kathryn M. Rexrode, Nancy R. Cook, Peter Kraft, Barbara B. Cochrane, Rowan T. Chlebowski, Gloria Y. F. Ho, Shumin M. Zhang

**Affiliations:** 1 Division of Preventive Medicine, Brigham and Women's Hospital and Harvard Medical School, Boston, Massachusetts, United States of America; 2 Department of Epidemiology, Harvard School of Public Health, Boston, Massachusetts, United States of America; 3 de Tornyay Center for Healthy Aging, University of Washington, Seattle, Washington, United States of America; 4 Department of Epidemiology and Population Health, Albert Einstein College of Medicine, Bronx, New York, New York, United States of America; 5 Department of Medicine, Los Angeles Biomedical Research Institute at Harbor-UCLA Medical Center, Torrance, California, United States of America; Centers for Disease Control and Prevention, United States of America

## Abstract

**Background:**

Estrogen and androgen have been linked to the regulation of circulating hepatocyte growth factor (HGF), an adipose tissue-derived cytokine. It is possible that the *CYP19A1* gene which alters sex hormones production may influence HGF levels. We examined the association between the *CYP19A1* gene variants and plasma HGF concentrations.

**Design:**

We evaluated 45 common and putative functional variants of *CYP19A1* and circulating levels of HGF among 260 postmenopausal women who later developed colorectal cancer from the Women's Health Initiative Observational Cohort. As the distribution of HGF levels was highly skewed, we transformed HGF concentrations for all women into a log-, ranked-, or normal score-scale value. Multiple linear regression with adjustment for age was used to evaluate the associations.

**Results:**

We observed an association between the rs7172156, rs1008805, rs6493494, rs749292, and rs11636639 variants and HGF levels in ranked and normal score scales (corrected p values ≤0.02), although the association of these 5 SNPs with log-scale HGF was not significant (corrected p values ≥0.16). The associations remained unchanged after additional adjustment for hormone therapy use and estradiol levels. These 5 SNPs, which were in linkage disequilibrium (pairwise *D′*≥97%, r^2^≥56%), constituted a block with 2 common haplotypes accounting for 82% frequency. The most common haplotype, TCCCA, was associated with lower ranked- or normal score-transformed HGF levels (corrected p values ≤0.001), whereas the second most common haplotype, CTTCA, was associated with higher ranked- or normal score-transformed HGF levels (corrected p values ≤0.02).

**Conclusion:**

Our findings of a potential association between the *CYP19A1* variants and circulating HGF levels warrant confirmation in studies with larger sample size.

## Introduction

Obesity contributes to the development of a variety of common chronic diseases among postmenopausal women, including cardiometabolic diseases and several cancers. Accumulation of adipose tissue as a result of excess body weight is associated with an increase of secretion of adipocytokines and growth factors including tumor necrosis factor alpha (TNF-α), interleukins (ILs), and insulin-like growth factor 1 (IGF1) [1]–[3]. Circulating hepatocyte growth factor (HGF), a less-studied adipokine to date, has been found to be elevated >3-fold in obese individuals than in those of normal body size [4]. Circulating HGF levels are also positively associated with risk for obesity-related clinical outcomes including hypertension [5], type 2 diabetes [6], and ischemic stroke [7]. It has been shown that HGF acts as an angiogenic growth factor in an autocrine and paracrine fashion in various cell types to promote cell migration, proliferation, invasion, and blood vessel growth [8].

Although it is clear that excess adiposity elevates HGF levels, the mechanisms underlying the regulation of HGF levels remain unknown. Experimental studies in animals and cell lines have shown that HGF expression are elevated by sex steroids including estradiol and testosterone [9]–[11] and the activation of HGF pathway is regulated by estrogen and androgen signaling [12], [13]. Aromatase is the key enzyme in estrogen biosynthesis which converts androstenedione to estrone and testosterone to estradiol [14]. Excess adiposity has been linked to increased aromatase activity, and, thus, increased estrogen production among postmenopausal women [15]. It is further possible that elevated aromatase activity, resulting from excess adiposity, alters sex steroids production which leads to changes in HGF levels.

Recent familial aggregation studies have reported significant parent-offspring and sibling correlations with HGF levels, independent of age and obesity-related phenotypes, suggesting that HGF secretion is also under genetic control [16]. Previous studies have shown that circulating HGF levels are associated with several tagging SNPs of the *HGF* gene [17]. Additionally, HGF levels are regulated by genes containing cis-acting elements including activating protein-1 (AP1), specificity protein 1 (SP1), and interferon regulatory factor-1 (IRF-1), and GATA [18]. *CYP19A1*, which encodes the aromatase enzyme and resides on chromosome 15q21.1, is one such gene that contains several of these regulatory elements (eg, AP1, SP1) in its promoter region [19], [20]. Given also that variation of the *CYP19A1* gene is associated with circulating estradiol and testosterone levels [21]–[23] which likely affect HGF levels, it is possible that genetic variants of the *CYP19A1* gene may be linked to HGF levels.

To test the genetic association between the aromatase enzyme and circulating HGF levels, we, therefore, conducted an evaluation of common and putative functional variants in the *CYP19A1* gene in relation to circulating HGF levels among postmenopausal women.

## Results

**Figure 1:**
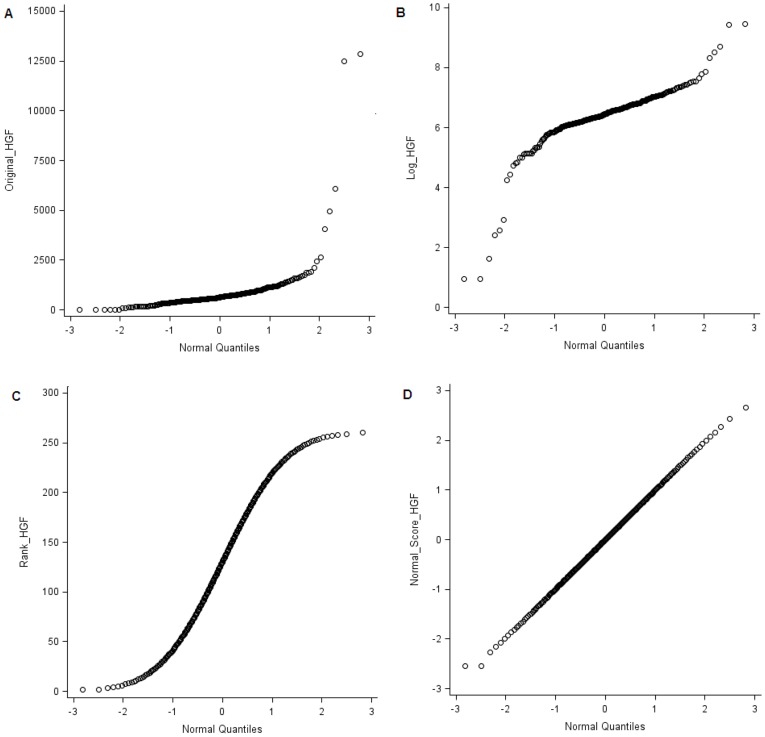
QQ plots of original (A), log- (B), ranked- (C), and normal score-transformed (D) HGF levels against normal quantiles.

We first evaluated the distribution of transformed HGF levels by plotting each of the transformed HGF levels against normal quantiles (Figure 1). The distribution of HGF appeared to be improved after each transformation. As can be seen from Figure 1B, some extreme values were still seen at both ends in the log-transformed HGF distribution. The outliers were somewhat improved in the ranked distribution (Figure 1C), and then resolved in the normal score-transformed distribution (Figure 1D). When we tested the 3 transformed distribution with the Shapiro-Wilk test, only normal score-transformed distribution had a non-significant p value of >0.05.

Two of the 47 SNPs were removed because of an MAF of <5% (Table S1). We also excluded 22 individuals who were missing >20% of genotype data and 1 with missing HGF levels, resulting in a total of 260 women in the present analysis. Circulating HGF levels were modestly correlated with body mass index (BMI) and cytokines of TNF-α and IL-6 (Spearman correlation coefficients were between 0.2 and 0.3, p values ≤0.005). In addition, circulating HGF was not associated with estradiol and total IGF1 levels in either users or nonusers of hormone therapy (HT) (Spearman correlation coefficients ≤0.10, p values ≥0.36), although levels of estradiol and IGF1 were each significantly different between HT users and nonusers [24]. Table 1 presents the selected characteristics according to the tertile distribution for HGF levels. Women in the highest group of circulating HGF were more likely to be obese and have higher plasma levels of TNF-α and IL-6, but were less likely to receive current hormone therapy. Circulating levels of estradiol and total or free IGF1 appeared to be similar across HGF groups in either users or non-users of HT. The estradiol levels in the 3 HGF groups were 47.0, 40.1, and 49.8 (p value = 0.49) among HT users, and 11.7, 12.8, and 13.3 (p value = 0.29) among nonusers. The total IGF levels in the HGF tertiles were 109.7, 118.6, and 95.3 (p value = 0.77) among HT users, and 145.8, 138.3, and 145.8 (p value = 0.80) among nonusers.

**Table 1:** Selected characteristics (mean ± standard deviation or %) among women from the Women's Health Initiative Observational Cohort Study (WHI-OS) according to plasma levels of hepatocyte growth factor (HGF).

	HGF*			
	Low (N = 86)	Medium (N = 87)	High (N = 87)	*p-value*
Median HGF (range), pg/mL	364 (3–499)	629 (500–791)	1119 (796–12857)	
Age, year	65.6 (6.9)	64.9 (7.5)	66.7 (6.5)	0.30
BMI, kg/m^2^	27.1 (4.7)	27.5 (5.0)	29.7 (6.1)	0.002
Diabetes, %	3.5	5.8	10.3	0.18
Current hormone therapy use, %	54.1	49.4	25.6	<0.001
IL-6, pg/mL	1.8 (1.5)	1.7 (1.1)	3.0 (2.7)	<0.001
TNF-α, pg/mL	2.5 (1.2)	2.7 (1.6)	7.8 (11.1)	0.001
Estradiol, pg/mL**	11.7 (4.2)	13.2 (8.9)	13.3 (7.7)	0.26
Total IGF1, ng/mL**	142.4 (41.4)	145.7 (56.7)	142.5 (50.4)	0.39
Free IGF1, ng/mL**	0.5 (0.3)	0.5 (0.4)	0.5 (0.4)	0.99

* According to tertile distribution.

** Among non-users of hormone therapy (N = 147).

Among the 45 variants evaluated, we found that six variants residing in the intronic region were associated with log-, ranked-, and normal score-transformed HGF levels with a pointwise p value of <0.05. When multiple comparisons were accounted for, 5 among them remained significantly associated with both ranked- and normal-scale HGF values (p values ≤0.04) (Table 2). Specifically, each additional copy of the minor allele of rs7172156 (T) and rs1008805 (C) was associated with lower mean HGF levels (Table 2). In contrast, the minor alleles of rs6493494 (T), rs749292 (T), and rs11636639 (C) were each associated with higher HGF levels (Table 2). The associations remained unchanged when we additionally adjusted for estradiol levels and current use of HT (data not shown). The associations were slightly attenuated with additional adjustment for BMI and circulating levels of TNF-α, and IL-6; yet rs7172156, rs1008805, and rs6493494 remained significantly associated with ranked- and normal-scale HGF after multiple-comparison corrections (p≤0.04). In the subgroup analysis according to HT use, the 5 observed SNPs were each associated with the ranked- and normal score-transformed HGF levels in either one of the HT groups, but none of these SNPs remained significant after correction of multiple comparisons (corrected p values ≥0.07).

**Table 2:** Association of the *CYP19A1* variants (A) and haplotypes (B) with plasma levels of hepatoacyte growth factors (HGF) in the Women's Health Initiative Observational Cohort Study (WHI-OS).

(A). Variants	Minor/Major allele	MAF^a^	Scale	β (95% CI)^b^	r^2^ b	*p_value_* c	*corrected p_value_* d
rs7172156	T/C	0.42	Log	−0.24 (−0.41,−0.07)	0.030	0.005	0.159
rs1008805	C/T	0.44	Log	−0.23 (−0.39,−0.06)	0.027	0.010	0.226
rs6493494	T/C	0.41	Log	0.20 (0.03,0.38)	0.021	0.018	0.398
rs749492	T/C	0.42	Log	0.20 (0.02,0.37)	0.020	0.026	0.486
rs11636639	C/A	0.42	Log	0.22 (0.05,0.38)	0.027	0.009	0.233
rs7172156	T/C	0.42	Ranked	−27 (−41, −14)	0.061	0.0002	0.003
rs1008805	C/T	0.44	Ranked	−27 (−40,−14)	0.060	0.0001	0.004
rs6493494	T/C	0.41	Ranked	27 (14,40)	0.062	0.0001	0.002
rs749492	T/C	0.42	Ranked	26 (13,39)	0.063	0.0002	0.003
rs11636639	C/A	0.42	Ranked	25 (12,38)	0.057	0.0004	0.006
rs7172156	T/C	0.42	Normal score	−0.33 (−0.51,−0.16)	0.055	0.00001	0.007
rs1008805	C/T	0.44	Normal score	−0.32 (−0.49,−0.14)	0.052	0.0007	0.013
rs6493494	T/C	0.41	Normal score	0.30 (0.13,0.47)	0.051	0.0004	0.011
rs749492	T/C	0.42	Normal score	0.28 (0.12,0.45)	0.050	0.0003	0.018
rs11636639	C/A	0.42	Normal score	0.28 (0.11,0.45)	0.047	0.0007	0.024

(B). Haplotype^e^		Freq		β (95% CI)^b^	r^2^ b	*p_value_* c	*corrected p_value_* d
CTTTC		0.40	Log	0.19 (0.02,0.36)	0.009	0.03	0.58
TCCCA		0.42	Log	−0.24 (−0.41,−0.06)	0.014	0.007	0.23
CCCCA		0.03	Log	0.27 (−0.28,0.80)	0.002	0.42	>0.99
CTCCA		0.13	Log	0.07 (−0.18,0.32)	0.0006	0.64	>0.99
CTCTC		0.01	Log	−0.10 (−0.83,0.63)	0.0001	0.77	>0.99
CTTTC		0.40	Ranked	25.2 (12.2,38.3)	0.027	0.0003	0.004
TCCCA		0.42	Ranked	−26.8 (−39.8,−13.9)	0.031	0.0001	0.001
CCCCA		0.03	Ranked	19.8 (−21.7,61.2)	0.002	0.40	>0.99
CTCCA		0.13	Ranked	2.0 (−17.4,21.3)	0.0001	0.95	>0.99
CTCTC		0.01	Ranked	0.04 (−56.1,56.3)	<0.0001	0.99	>0.99
CTTTC		0.40	Normal score	0.30 (0.13,0.47)	0.022	0.001	0.02
TCCCA		0.42	Normal score	−0.33 (−0.50,−0.16)	0.027	0.0001	0.005
CCCCA		0.03	Normal score	0.28 (−0.27,0.82)	0.002	0.38	>0.99
CTCCA		0.13	Normal score	0.05 (−0.21,0.30)	0.0003	0.81	>0.99
CTCTC		0.01	Normal score	−0.07 (−0.81,0.66)	0.0001	0.86	>0.99

a MAF = minor allele frequency.

b Adjusted for age.

c Pointwise 10,000 permutation tests.

d Familywise 10,000 permutation tests.

e Based on the 5 variants in (A).

**Figure 2:**
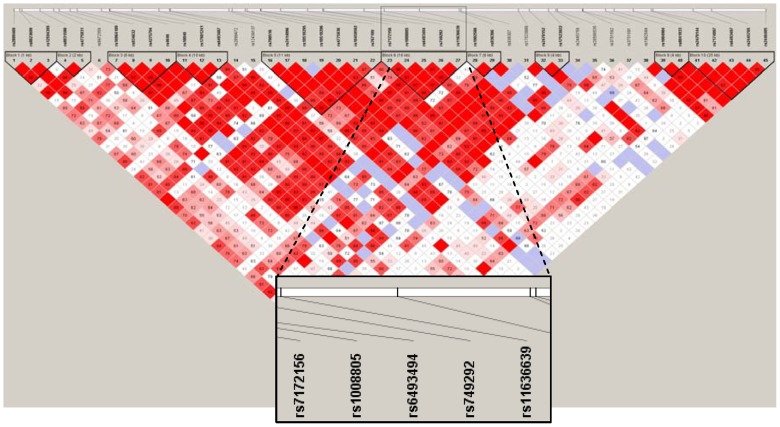
Linkage disequilibrium (LD) plot of the CYP19A1 gene in the Women Health Initiative Observational Study (WHI-OS) cohort. LD is based on the measures of D′ and LOD (bright red, D′ = 1 and LOD≥2; shades of pink/red, D′<1 and LOD≥2; white, D′<1 and LOD<2; blue, D′ = 1 and LOD<2). Block #6 (highlighted) contains the 5 SNPs with strongest associations with circulating hepatocyte growth factor (HGF) levels.

The 5 variants, which spanned approximately 17 kb, were in linkage disequilibrium (pairwise *D′*≥97%, r^2^≥56%). None of these 5 SNPs remained significant when all of them were included in the multiple regression model (p value ≥0.15). The 5 variants constituted block #6 of the *CYP19A1* gene with 2 common haplotypes accounting for 82% frequency (Figure 2). The first common haplotype (TCCCA, 42%) containing the minor alleles of rs7172156 (T) and rs1008805 (C) was significantly associated with lower HGF levels in either ranked- or normal score-transformations (corrected p value ≤0.001), whereas the other haplotype (CTTTC, 40%) with the minor alleles of rs6493494 (T), rs749292 (T), and rs11636639 (C) was associated with higher ranked or normal score-transformed HGF levels (corrected p value ≤0.02, Table 2). In addition, rs7172156 appeared to be the tagging SNP for the TCCCA haplotype with its minor allele (T) having similar frequency and risk estimate to the haplotype. For the CTTTC haplotype, the rs6493494 was the most plausible SNP whose allele (T) reflected the frequency and risk estimate for the haplotype.

## Discussion

In this exploratory analysis, we evaluated the association between variants in the *CYP19A1* gene, a sex steroid synthesizer, and plasma levels of a less known adipokine, HGF. The skewness of HGF distribution was reduced through the transformation into log, ranked, and normal score scales, which were each regressed on the gene variants. We found that the ranked and normal-scale HGF concentrations performed better than the log-scale HGF. Five of the *CYP19A1* gene variants, rs7172156, rs1008805, rs6493494, rs749292, and rs11636639 were each associated with ranked- and normal-scale HGF. However, the association of these 5 SNPs with log-scale HGF levels was not significant after correction for multiple comparisons.

Our findings are the first observational evidence showing an association between the *CYP19A1* gene and HGF levels. One possible explanation for the association may be due to the regulation of the aromatase enzyme on HGF secretion. However, we did not observe an association between the rs700518 SNP which has been linked to aromatase activity in adipose cells [25] and HGF levels. Alternatively, estrogen and/or androgen levels regulated by the aromatase enzyme may mediate the observed association. In a large study of postmenopausal women, Haiman et al. reported that the minor alleles of the two tagging SNPs, rs749292 and rs727479, are associated with a 10%–20% increase in endogenous estradiol levels in postmenopausal women [21]. In this study population of women not using hormone therapy, we did not observe an association between HGF and endogenous estradiol levels. We also did not find an association between rs749292 and estradiol levels, although rs6493494 and rs11636639, two other *CYP19A1* variants residing in the same haplotype block in which rs749292 is located, were weakly associated with circulating estradiol at pointwise significant level (uncorrected p values ≤0.04). The low statistical power (N = 147) may explain the null association among *CYP19A1*, estradiol, and HGF levels. Additionally, androgen levels or the ratio of estrogen over androgen levels may contribute more than estrogen alone to the association. Of the observed SNPs that were associated with HGF levels, the rs1008805 SNP has been linked to circulating testosterone levels in women [23], suggesting a potential role of androgen in mediating the association. Future studies are warranted to test the androgen hypothesis.

Previous studies have suggested that the rs749292 variant is associated with risk for developing female hormone-associated cancers. A study has reported an elevated risk of ovarian cancer among Caucasian and Japanese postmenopausal women who carried both copies of the minor allele (T) of the rs749292 variant [26]. The other consortium study has also reported an increased risk for endometrial cancer among Caucasian women carrying the rs749292 T allele [27]. Both studies have attributed the genetic association to elevated estradiol concentrations. HGF is an angiogenic growth factor which promotes cell migration, proliferation, and invasion [8], and circulating HGF levels tend to be higher among patients with established cancers [28]–[30]. Our findings of the potential association between *CYP19A1* including the rs749292 variant and HGF levels suggest that *CYP19A1* is also likely associated with cancer progression due to elevated HGF levels. Our findings in women who later developed colorectal cancer also suggest that the observed association between CYP19A1 and HGF levels may be more relevant to cancer population. However, our analysis is exploratory and studies with larger sample size are needed to confirm the association.

Limitations of this study include the small sample size which likely resulted in the skewness of the HGF distribution and invalidated the use of statistics such as mean and standard deviation. Accordingly, we have first conducted the conventionally used log-scale transformation to reduce the skewed distribution of HGF. Despite the improvement, the deviation from normality test for the log- and ranked-transformed HGF distribution remained significant (p<0.001). We, thus, additionally transformed HGF concentrations into normal scales to achieve symmetrical and normally shaped distribution. The small sample size also likely resulted in the null correlation between HGF (either transformed or nontransformed) and endogenous estradiol levels among postmenopausal women in whom estradiol levels tend to be very low. Given also the null association between the CYP19A1 gene and endogenous estradiol levels from our study and the other small study of 345 postmenopausal women [31], a study with larger sample size such as the one by Haiman and colleagues (>3100 women) [21] may be required for detecting the association. Alternatively, given that endogenous estradiol is produced through conversion from androgens in postmenopausal women, it is possible that androgen levels or the ratio of estrogens over androgens are also relevant to HGF levels. The lack of androgen data in this study does not allow for assessment of the mediating role of androgen in the observed association. Finally, the present analysis was performed among women who later developed colorectal cancer. Thus, the findings may not be applicable to the general population, although both *CYP19A1* variants and HGF levels were not strongly associated with colorectal cancer risk [32], [33].

### Conclusion

In summary, our findings of an association between the *CYP19A1* gene variants and circulating HGF levels offer a novel mechanism underlying the link between obesity and endocrine-related diseases. Additionally, the CYP19A1 gene may be relevant to cancer progression, given its potential role in regulating HGF which has angiogenic and mitogenic properties. However, our findings are exploratory and require confirmation in larger observational studies.

## Materials and Methods

### Study population

The Women's Health Initiative Observational Study (WHI-OS) is a large, multifaceted study of 93,676 postmenopausal women aged 50 to 79 years who were recruited between October 1, 1993 and December 31, 1998 [34]. At baseline, women provided informed consent and completed questionnaires and a physical examination. Fasting blood samples were collected, centrifuged, frozen on site at −70°C, and stored in the specimen repository. In the present analysis, we included a total of 283 women of European ancestry with available data on the *CYP19A1* gene variants and HGF levels from two previous studies [32], [33]. These 283 women had later developed colorectal cancer after blood DNA was collected.

### HGF biochemical assay

The measurement of plasma HGF levels has been previously described [35]. Briefly, HGF levels were measured by a multiplex assay based on Luminex xMAP technology. The interassay coefficient of variation was 11.7% and the 3-year intraindividual correlation coefficient was 0.91 [35].

### Selection of single nucleotide polymorphism (SNP) and genotyping

Selection and genotyping of the *CYP19A1* variants has been performed and described previously [32]. Briefly, we first included 42 tagging SNPs that capture common variation and linkage disequilibrium (LD) structure within the gene region as well as 20 kb upstream and downstream of the gene based on the study from the Breast and Prostate Cohort Consortium project (BPC3) and additionally through the identification by the Tagger program [36] using the National Center for Biotechnology Information Build 35 assembly. Selection of the tagging SNPs was based on a haplotype frequency (*R*_h_^2^) of ≥0.70 between observed haplotypes and those predicted based on tagging SNPs (used in the BPC3 project) or a pairwise correlation coefficient (r^2^) of ≥0.70 between tagging SNPs and untyped SNPs (used in the Tagger program), and a MAF of ≥1% in the Caucasian populations. We also included 5 putative functional SNPs obtained through literature search. All together, there were 47 SNPs selected for this gene.

DNA was extracted from the buffy coat fraction of centrifuged blood using the QIAmp Blood Kit (Qiagen, Chatsworth, CA) and genotyping determination was performed with the Sequenom MassARRAY Genotyping system. Quality control of Sequenom Genotyping was carried out by repeating the genotyping on 66 duplicate samples with an average concordance rate of 99.7%. The average genotyping drop-out rates were 6.8%, ranged from 3.1% to 16.9% (Table S1).

In the present analysis, SNPs were excluded from further analysis if they had a MAF of <5% or deviated from Hardy-Weinberg equilibrium (HWE) among all women (p<0.001). We also excluded individuals who were missing >20% of genotype data.

### Statistical Analysis

Previous studies of HGF levels and health outcomes in the general population have treated HGF as a categorical variable [37]–[39], which avoids problems derived from skewness or extreme values of the distribution. As the present analysis was based on continuous HGF levels in which distribution was highly skewed (p value for normality <0.001), we transformed HGF concentrations for all women into a log-, ranked-, or normal score (van der Waerden (VW) normal score method)-scale value. The practice of transforming non-normal raw data into a near-normal form is usually adopted when data are not normally distributed [40]. The association between the *CYP19A1* gene variants and the transformed HGF concentrations were then assessed through a linear regression as implemented in PLINK [41] with an additive scoring for 0, 1, or 2 copies of the minor allele of each SNP. The analyzed models were adjusted for age (continuous). An empirical p value was calculated that gave a pointwise estimate of the significance level of each SNP.

Haplotype LD blocks were constructed using the option of confidence interval on D′ implemented in Haploview. Haplotype frequency and expected haplotypes for each subject were then inferred based on the unphased genotype data using the expectation-maximization (EM) algorithm in PLINK. We used linear regression to estimate haplotype-specific association with the rest of the haplotypes as the referents. Haplotypes with estimated frequency of <1% were excluded from the analysis. To control for comparisons for multiple SNPs (or haplotypes), we performed 10,000 permutations to generate a gene-specific (familywise) empirical p value for each SNP (or haplotype) and to determine how frequently the identified association would occur by chance. We performed these permutations using the max(T) permutation option in PLINK.

## Supporting Information

Table S1. (DOC)
