# Supplementary material for: The Aromatase Gene (CYP19A1) Variants and Circulating Hepatocyte Growth Factor in Postmenopausal Women
Source: PLoS One. 2012 Jul 25;7(7):e42079. doi: 10.1371/journal.pone.0042079 (PMC3405042; doi:10.1371/journal.pone.0042079)
Supplement: Table S1. — (DOC) [file pone.0042079.s001.doc]

Table S1. Characteristics of the *CYP19A1* variants.

| rsname | a1 | a2 | Minor allele frequency | Hardy-Weinberg p value | % missing genotypes |
| --- | --- | --- | --- | --- | --- |
| rs2899469 | G | A | 0.44 | 0.37 | 8.44 |
| rs8023699 | C | A | 0.18 | 1.00 | 3.44 |
| rs12594395 | G | C | 0.40 | 0.51 | 8.13 |
| rs8031580 | A | G | 0.45 | 0.80 | 5.63 |
| rs4775931 | A | T | 0.27 | 1.00 | 7.19 |
| rs9972359 | G | A | 0.40 | 0.24 | 6.88 |
| rs16964189 | A | G | 0.24 | 0.73 | 5.00 |
| rs934632 | T | C | 0.18 | 0.29 | 3.13 |
| rs4275794 | G | A | 0.24 | 0.73 | 5.94 |
| rs4646 | T | G | 0.28 | 0.87 | 9.06 |
| rs10046 | T | C | 0.48 | 0.90 | 5.00 |
| rs17601241 | T | C | 0.09 | 0.70 | 7.19 |
| rs6493487 | C | T | 0.28 | 0.87 | 8.75 |
| rs28757184* | T | C | 0.03 | 1.00 | 4.69 |
| rs2899472 | T | G | 0.24 | 0.73 | 3.75 |
| rs12439137 | C | T | 0.13 | 1.00 | 8.75 |
| rs700518 | G | A | 0.48 | 0.46 | 3.75 |
| rs2414096 | T | C | 0.46 | 0.89 | 16.88 |
| rs10519295 | G | A | 0.10 | 1.00 | 3.75 |
| rs10519296 | G | C | 0.05 | 0.51 | 4.06 |
| rs4775936 | A | G | 0.45 | 0.61 | 6.25 |
| rs10459592 | A | C | 0.45 | 0.30 | 10.00 |
| rs767199 | T | C | 0.46 | 0.80 | 8.44 |
| rs7172156 | T | C | 0.42 | 0.37 | 5.94 |
| rs1008805 | C | T | 0.44 | 0.31 | 5.94 |
| rs6493494 | T | C | 0.41 | 1.00 | 3.13 |
| rs749292 | T | C | 0.42 | 0.30 | 8.44 |
| rs11636639 | C | A | 0.42 | 0.36 | 7.19 |
| rs1902586 | T | C | 0.07 | 1.00 | 9.06 |
| rs936306 | A | G | 0.16 | 1.00 | 6.56 |
| rs936307 | T | G | 0.08 | 0.21 | 4.69 |
| rs17523880 | T | G | 0.13 | 0.25 | 8.75 |
| rs2470152 | C | T | 0.50 | 0.71 | 3.13 |
| rs17523922 | C | G | 0.11 | 0.75 | 5.63 |
| rs2445759 | A | C | 0.06 | 0.06 | 8.75 |
| rs28566535 | C | A | 0.07 | 0.35 | 7.81 |
| rs3751592 | G | A | 0.33 | 0.31 | 9.38 |
| rs3751591 | C | T | 0.17 | 0.11 | 4.06 |
| rs1902584 | A | T | 0.10 | 1.00 | 9.06 |
| rs1004984 | T | C | 0.39 | 0.90 | 3.75 |
| rs28757082* | A | C | 0.001 | 1.00 | 7.50 |
| rs8041933 | T | C | 0.22 | 0.58 | 10.00 |
| rs2470144 | G | A | 0.47 | 0.80 | 5.63 |
| rs7174997 | A | C | 0.20 | 1.00 | 8.13 |
| rs6493497 | T | C | 0.13 | 0.39 | 8.44 |
| rs2445765 | G | C | 0.17 | 0.50 | 6.25 |
| rs2446405 | T | A | 0.16 | 0.47 | 8.75 |

*. Excluded from the analysis.
